# The role of vitamin D in pre-eclampsia: a systematic review

**DOI:** 10.1186/s12884-017-1408-3

**Published:** 2017-07-15

**Authors:** Juhi M. Purswani, Pooja Gala, Pratibha Dwarkanath, Heather M. Larkin, Anura Kurpad, Saurabh Mehta

**Affiliations:** 1 Division of Nutritional Sciences, Cornell University, 314 Savage Hall, Ithaca, NY 14853 USA; 2 Weill-Cornell Medical College, New York, NY USA; 3 St. John’s Research Institute, Bangalore, Karnataka India

**Keywords:** Vitamin D, Pregnancy, Pregnancy-induced hypertension, Pre-eclampsia, Endothelial function

## Abstract

**Background:**

The etiology of pre-eclampsia (PE) is not yet fully understood, though current literature indicates an upregulation of inflammatory mediators produced by the placenta as a potential causal mechanism. Vitamin D is known to have anti-inflammatory properties and there is evidence of an inverse relationship between dietary calcium intake and the incidence of PE. Evidence of the role of vitamin D status and supplementation in the etiology and prevention of PE is reviewed in this article along with identification of research gaps to inform future studies.

**Methods:**

We conducted a structured literature search using MEDLINE electronic databases to identify published studies until February 2015. These sources were retrieved, collected, indexed, and assessed for availability of pregnancy-related data on PE and vitamin D.

**Results:**

Several case-control studies and cross-sectional studies have shown an association between vitamin D status and PE, although evidence has been inconsistent. Clinical trials to date have been unable to show an independent effect of vitamin D supplementation in preventing PE.

**Conclusions:**

The included clinical trials do not show an independent effect of vitamin D supplementation in preventing PE; however, issues with dose, timing, and duration of supplementation have not been completely addressed.

**Electronic supplementary material:**

The online version of this article (doi:10.1186/s12884-017-1408-3) contains supplementary material, which is available to authorized users.

## Background

Each day, 830 women die from preventable pregnancy-related causes; low- and middle-income countries bear the greatest burden of disease [1]. Hypertensive disorders of pregnancy, including gestational hypertension, pre-eclampsia (PE), and eclampsia, are among the major complications that account for approximately 14% of maternal mortality [1, 2]. Pregnancy induced hypertension, defined as blood pressure greater than 140/90 mmHg on two consecutive occasions ≥6 h apart occurring after 20 weeks of pregnancy, complicates approximately 10% of all pregnancies worldwide. Pre-eclampsia (PE) is hypertension and proteinuria (protein in urine ≥0.3 g/24 h (1+ dipstick) on two occasions ≥6 h apart) or edema [3, 4]. It is a major cause of maternal and perinatal morbidity and mortality and complicates 2% to 8% of pregnancies [3, 5]. Early onset severe PE (EOSPE) is diagnosed between 20 to 34 weeks gestation and is associated with a 20-fold increased risk for maternal mortality compared to PE after 34 weeks gestation [6] called late onset severe PE (LOSPE). Pregnant women who show signs of pregnancy induced hypertension or PE can develop eclampsia, which is the occurrence of unexplained seizures [7].

The complications of PE include eclampsia, disseminated intravascular coagulation and the HELLP syndrome (hemolytic anemia, elevated liver enzymes, and low platelets [5]). Risks to the fetus include intrauterine growth restriction (IUGR) and fetal death [8]. Pathognomonic features shared by PE and IUGR include abnormal placental implantation and reduced trophoblastic invasion [9]. Similarly, EOSPE and LOSPE are associated with fetal death, perinatal death, and severe neonatal morbidity. For example, in an analysis of 456,668 singleton births in Washington state, EOSPE was associated with high risk of fetal death; the adjusted odds ratio (aOR) was 5.8 (95% Confidence Interval (CI): 4.0–8.3 vs. 1.3 for LOSPE (95% CI: 0.8–2.0). The aOR for perinatal death/severe neonatal morbidity was 16.4 (95% CI: 14.5–18.6) in EOSPE and 2.0 (95% CI: 1.8–2.3) in LOSPE [10]. Women with a history of PE also have a higher risk of cardiovascular disease later in life [11, 12].

For years, PE has been hypothesized to be a two-stage disorder [13]. In the first stage, placental perfusion is reduced resulting in defective placental implantation. In the second stage, reduced vascularization at the placental site activates a maternal inflammatory response. This leads to generalized endothelial dysfunction and the release of excessive anti-angiogenic factors into the maternal bloodstream resulting in hypertension. In PE, higher amounts of soluble fms-like tyrosine kinase 1 (sFLT-1) are produced in the placenta. sFLT-1 competitively binds to placental growth factor (PIGF) and vascular endothelial growth factor (VEGF) creating an angiogenically imbalanced vascular environment that prevents proper endothelial preservation [14, 15]. More recently, a modified version of the two-stage hypothesis has been developed, which proposes that maternal constitutional factors (genetics, obesity, diet, co-morbid disease) in combination with normal inflammatory changes in pregnancy can lead directly to endothelial dysfunction with or without the contribution of reduced placental perfusion [16, 17].

Hypovitaminosis D has been associated with PE [18]. While the pathogenesis of PE involves a number of biological processes, there are several hypotheses to suggest how vitamin D levels may affect these processes (Table 1). These include vitamin D’s role in modulating pro-inflammatory responses and decreasing oxidative stress in PE, promoting angiogenesis through VEGF and gene modulation, and decreasing blood pressure through the renin-angiotensin system (RAS) [19–25].

**Table 1:** Summary of Effects of increased vitamin D on the pathogenesis of pre-eclampsia (PE)

Stage of PE	Characteristic of PE	↑ Vitamin D
Stage 1	Inflammation-linked abnormal placental implantation	↓ Predisposition to pro-inflammatory response [20]
		↑ Regulation of genes associated with placental invasion and normal implantation [20]
Stage 2	Vascular Endothelial dysfunction	↑ Vascular structure, elasticity and intima-media thickness
		↓ Blood pressure (regulation of renin-angiotension system) [63]
		↓Oxidative stress [24]
	Proteinuria mediated by renal vascular endothelial growth factor (VEGF)	↑ Vascular smooth muscle cell proliferation by increasing VEGF gene transcription [19]

### Biological plausibility of the role of vitamin D in PE

Vitamin D_3_, or cholecalciferol, is formed endogenously when 7-dehydrocholesterol in keratinocytes is converted to a seco-steroid pro-hormone upon irradiation by UV-B light. This is followed by two successive hydroxylations - first at the 25 position to form 25-hydroxyvitamin D [25(OH)D], which occurs in the liver, and second to form the active hormonal metabolite 1,25-dihydroxyvitamin D [1,25(OH)_2_D], also called calcitriol, which is largely carried out in the kidney. Calcitriol binds to its cognate nuclear receptor and modulates gene expression mainly related to calcium absorption in the intestine. Calcitriol can also modulate immune function through a rapid action pathway by binding to receptors on the plasma membrane when the active hormone is synthesized in situ by several extra-renal tissues, namely macrophages, endothelium, cells in the prostate, and keratinocytes, as all of them express the vitamin D receptor [25].

Vitamin D status is determined by the measurement of its circulating form, 25-hydroxyvitamin D [25(OH)D] [26]. Vitamin D is considered adequate when 25(OH)D levels are above 50 nmol/L, as defined by the Institute of Medicine. A level between 30 and 50 nmol/L is considered insufficient, and less than 30 nmol/L, deficient [27].

In the past decade, maternal vitamin D insufficiency and deficiency have increasingly been recognized as a public health concern. Insufficiency has been linked to adverse maternal and fetal outcomes, including poor fetal and infant bone mineralization [28, 29], hypocalcemia and rickets in neonates [30]. A number of prospective observational studies have shown a high prevalence of hypovitaminosis D during pregnancy in developing and developed countries [31]. Risk factors that affect vitamin D status include season, time of the day, latitude, clothing and skin color [32]. Vitamin D deficiency is commonly found among pregnant women in various ethnic populations [33–37]. African American women of reproductive age have been found to be at particularly high risk for vitamin D deficiency in the United States [18]. Clothing with minimal skin exposure [38, 39], increased urbanization, skin pigmentation [40], and vegetarian diets [35] are all believed to have contributed to a vitamin D deficiency epidemic worldwide.

During the course of pregnancy, evidence from observational studies shows divergent data on the concentration of serum 25(OH)D levels in different trimesters of pregnancy, with either decline [41], increase [42], or absence of change in vitamin D levels with progression [43, 44].

It is plausible that a deficiency in vitamin D and its downstream products can play a role in the etiology of PE. Human decidual cells at the fetal-maternal interface synthesize active 1,25(OH)_2_D via 1alpha-hydroxlyase (CYP72B1) [45–47]. Syncytial trophoblasts responsible for invading the uterine wall for fetal implantation also express active CYP27B1, in addition to vitamin D receptor (VDR), vitamin D binding protein (VDBP), 25-hydroxylase, and 24-hydroxlyase. Metabolic homeostasis of these proteins has been shown to be significantly altered in placental tissue from pregnancies with PE compared to controls [48, 49]. In a study in which human extravillous trophoblasts were treated in vitro with 1,25(OH)_2_D or 25(OH)D, there was a significant increase in EVT invasion into cell cultures when compared with untreated controls (*p* < 0.01) [47].

Additionally, vitamin D likely plays a key role in the pathology of pre-eclamptic conditions by affecting blood pressure through calcium homeostasis and/or modulating inflammation and immunity, which are examined further in this review.

### Role of vitamin D as an anti-inflammatory agent and immune modulator

Vitamin D is thought to play a significant role in PE as an immune modulator [20, 50]. It may help mount an appropriate maternal immune response to the placenta preventing the release of anti-angiogenic factors into the bloodstream and modulating hypertension [50]. For example, 1,25(OH)_2_D suppresses T cell receptor-induced T cell proliferation, altering the cytokine expression profile and diminishing the production of γ-interferon and interleukin-2 [51]. 1,25(OH)_2_D down regulated pro-inflammatory cytokines, tumor necrosis factor-α, and interleukin-6 secretions (*p* < 0.05) in trophoblastic preparations from placentas of pre-eclamptic women collected after delivery and cultured in the presence of calcitriol compared to pre-eclamptic placentas cultured in the absence of 1,25(OH)_2_D [52]. In a study conducted on 100 normotensive and 100 pre-eclamptic women, both plasma vitamin D deficiency (OR 4.2, 95% CI: 1.4–12.8, *p* = 0.04) and interleukin-6 elevation (OR 4.4, 95% CI: 1.8–10.8, *p* < 0.01) were independently associated with PE. However there was no association between plasma vitamin D deficiency and interleukin-6 elevation [53].

1,25(OH)_2_D is also suspected to be involved in the regulation of IL-10, which has an inhibitory effect on pro-inflammatory cytokines expression in the human placenta. For example, 1,25(OH)_2_D may undertake the anti-inflammatory effects of IL-10 by itself to inhibit expression of placental Th1-cytokines, which are increased in PE. Barerra et al. showed that calcitriol down-regulates IL-10 under normal, natural and experimental inflammatory conditions in cultured human trophoblasts [54]. Calcitriol has also been shown to decrease TNF-a and IL-6 expression [52].

In addition to down-regulating the release of anti-angiogenic factors, vitamin D has been shown to promote angiogenesis in endothelial progenitor cells, by possibly increasing VEGF expression and pro-matrix metalloproteinase (pro-MMP-2) activity [55]. MMPs are implicated in the pathogenesis of vascular dysfunction associated with PE [56, 57]. There is evidence that 1,25(OH)_2_D is synthesized in the vascular endothelial cells [58] and induces vascular smooth muscle cell (VSMC) proliferation [19]. Brodowski et al. showed that supplemented 1,25(OH)_2_D on endothelial progenitor cells reversed endothelial dysfunction seen in preeclampsia [59]. Cardus et al. found that the effect of 1,25(OH)_2_D on VSMC proliferation is mediated by increased VEGF expression, while others find no relation between vitamin D and pro-angiogenic factors [15]. There is currently not enough data to strongly support the hypothesis that impaired angiogenesis explains the association between vitamin D deficiency and PE [60].

### Role of vitamin D and blood pressure regulation

An inverse relationship between plasma 1,25(OH)_2_D and plasma renin activity has been observed. The RAS plays an important role in regulation of blood pressure. During normal pregnancy, RAS is stimulated so that there is an increase in circulating levels of renin, angiotensinogen and angiotensin II [61]. In PE, circulating serum angiotensin I, angiotensin II and aldosterone are lower compared to normotensive women, while plasma active renin levels and autoantibodies to the Angiotensin II type 1 receptor, which stimulate receptor signaling to increase systemic blood pressure, are higher [62–64]. These findings suggest a hemodynamic dysregulation in PE involving the RAS. Vitamin D metabolites may suppress renin gene transcription by a vitamin D receptor (VDR) dependent pathway [65] or reduce autoantibodies to the Angiotensin II type I receptor [64]. In one study, oral vitamin D2 (VD2, 270 IU/day) and vitamin D3 (VD3, 15 IU/day) lowered mean arterial blood pressure (mm Hg) in pregnant rats infused with autoantibodies to Angiotensin II type I receptor (VD2: 105 +/−2, VD3: 109 +/− 2) versus rats that were infused with autoantibodies to Angiotensin II type I receptor but did not receive oral VD2 or VD3 (121 +/− 4) [64]. Studies have shown an inverse relationship between plasma 1,25(OH)_2_D levels and blood pressure [66], and plasma 1,25(OH)_2_D levels and essential hypertension [67]. In vitro and in vivo studies have found receptors for calcitriol in vascular smooth muscle [68] and heart muscle [69]. A study of mice found receptors for calcitriol in regions of the spinal cord and brain stem that are associated with blood pressure regulation [70].

### Role of vitamin D and vitamin D receptor (VDR) in calcium homeostasis

The effects of calcium supplementation on pregnancy-induced hypertensive disorders are well characterized. A Cochrane meta-analysis of 13 randomized trials involving 15,730 women found that at least 1 g daily calcium supplementation lowers the average risk of PE by approximately half (relative risk [RR]: 0.45, 95% CI: 0.31–0.65) [71]. Low calcium levels may lead to hypertension by stimulating either the parathyroid hormone (PTH) or renin release, resulting in vasoconstriction due to increased intracellular calcium in vascular smooth muscle [72, 73]. Further calcium absorption has been found to be positively associated with serum 1,25(OH)_2_D concentrations in late pregnancy [74, 75]. The high concentration of this metabolite inhibits serum PTH synthesis and secretion, promoting the active intestinal absorption of calcium.

It is also observed that calcium sufficiency may occur independently of vitamin D sufficiency. In a study of female mice with a VDR null mutation and rickets, intestinal absorption of calcium and whole-body mineral content was significantly reduced compared to wild type mice before pregnancy (0.381 ± 0.026 vs. 0.529 ± 0.023, *p* < 0.001). During pregnancy, 1,25(OH)_2_D levels doubled in both groups and VDR null mice gained 158% of bone mineral content from baseline. By day 16.5 of pregnancy, intestinal calcium absorption in VDR null mice was equivalent to non-pregnant wild type mice, but did not reach levels of pregnant wild type mice [76].

Given the role of vitamin D in modulation of inflammation and immune function, placental implantation, angiogenesis and adverse birth outcomes such as low birth weight and SGA, we examined the evidence that vitamin D may play a role in the etiology and prevention of PE in this manuscript.

## Methods

### Search strategy

The review protocol was designed a priori to answer the question, “What are the effects of vitamin D concentrations and supplementation during pregnancy on pre-eclampsia in women?” We conducted a literature search using MEDLINE electronic databases (via PubMed) to identify published studies until February 2015. Search terms: (preeclampsia OR pre-eclampsia) AND (vitamin D OR hypovitaminosis D OR 1,25 dihydroxyvitamin D OR (vitamin D AND (supplementation OR supplement)) OR dihydroxyvitamin D OR 25 hydroxyvitamin D). The search was confined to peer-reviewed articles that were published in English and contained an abstract. Reference lists of journal articles were also screened for additional citations fitting our search criteria.

Articles were screened independently by two authors (JP and PG) for relevance based on the title and abstract. In the event that the number of RCTs returned by the search was insufficient to conduct a meta-analysis of PIH outcomes, authors planned to give a structured review of both experimental and observational studies. Considering retrospective and observational designs are more prone to bias than experimental design, results of clinical trials are presented separately and given more weight in conclusions and interpretations. An individual Risk of Bias assessment was not done given structured review design. Selected journal articles were then retrieved, collected, indexed, and assessed for availability of pregnancy-related data on PE and vitamin D by JP and PG. The inclusion criteria for this review were availability of an abstract, clinical data on vitamin D concentrations and/or supplementation in association with PIH outcomes in any global setting, and subjects that included pregnant participants aged 18 years and above without other medical co-morbidities. Studies were excluded if they were reviews, commentaries, editorials, letters, non-human studies, didn’t include maternal vitamin D measured during gestation as a primary or secondary variable, or did not include PE as a primary or secondary outcome.

### Data extraction

Selected studies were read and desired data were extracted independently by two authors (JP and PG). A third author, PD cross-checked data extraction to establish 100% agreement between the two independent reviewers. The following elements were extracted for each study: Author; year of publication; country; study design; objective, methods, exposure and outcome measures, sample size and key findings. All authors reviewed the final summary of selected studies and resolved any data discrepancies through discussion. Principal summary measures included risk of PIH outcomes and change in means of continuous PIH indicators such as hypertension.

## Results

**Fig. 1:**
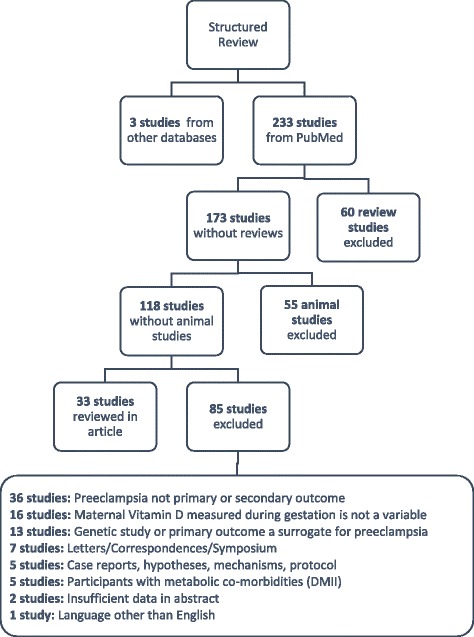
Search Flow. Detailed summary of the search process and protocol

The structured literature search resulted in 233 articles. 200 studies were excluded (60 reviews, 55 non-human studies, 85 studies that didn’t meet the inclusion criteria described above (Fig. 1). A total of 33 studies were extracted for further review. The reviewed studies included 3 cross-sectional studies, 20 case control studies, 2 retrospective cohort studies, 6 prospective cohort studies and 2 randomized controlled trials.

A detailed summary of the search strategy and results is presented in Fig. 1. The PRISMA Checklist for this review can be found in Additional file 1.

### Evidence to support the role of vitamin D in PE

Several cohort and case control studies measuring vitamin D before the development of PE show an association between vitamin D and PE (Tables 2 and 3). In a large cohort study of 22 057 nulliparous women, no association was found between vitamin D intake from diet alone and the incidence of PE. However, when considering total vitamin D intake (diet + supplement) of 15–20 μg/d compared to less than 5 μg/d, there was an adjusted OR of 0.76 (95% CI 0.60–0.95) for diagnosis with PE [77]. There was an adjusted OR of 0.72 (95% CI 0.58–0.92) in women with vitamin D intake of 10–15 μg/d from supplementation of vitamin D alone when compared with no supplementation. A limitation of this study was that it was unable to adjust for intake of long chain n-3 fatty acids, which correlated with vitamin D intake in a Norwegian diet. Another prospective cohort study of nulliparous women with singleton pregnancies found no significant association between vitamin D deficiency in the 1st trimester and risk for PE; however, at 24–26 weeks of gestation mean maternal 25(OH)D was significantly lower in women who developed PE compared with those who did not (*p* = 0.03) [60]. The adjusted OR was 3.24 for PE (95% CI:1.37–7.69) in women with 25(OH)D levels less than 50 nmol/l. Strengths of this study were that vitamin D was measured in both high and low risk women, representing a realistic clinical scenario. A retrospective cohort study showed that at entry to care (13.7 ± 5.7 weeks) there was an increased risk of PE in women with 25(OH)D levels less than 49.9 nmol/L and high parathyroid hormone (> 62 pg/mL) (aOR 2.86; 95% CI: 1.28–6.41) [78]. However, there was no association in women with vitamin D insufficiency who did not also have high PTH.

**Table 2:** Characteristics of included reports

Author, Location, Year	Design	Subjects	Time of vitamin D measurement
Cross-Sectional Studies			
August et al., USA, 1992 [88]	Cross-sectional	11 – women with PE	3rd trimester
		9 – chronic HTN	
		12 – normotensive pregnant controls	
Fernandez- Alonzo et al., Spain, 2012 [97]	Cross-sectional	466 – pregnant women (7 had PE)	1st trimester
Pena HR, et al. Brazil 2015 [83]	Cross-sectional	179 -- pregnant women recruited before delivery	Near delivery
Case Control Studies			
Abedi et al., Iran, 2014 [84]	Case Control	59 – women with PE	At delivery
		59 – healthy pregnant controls	
Achkar et al., Canada, 2015 [81]	Case Control	169 – women with PE	<20 weeks gestation
		1975 – healthy pregnant controls	
Anderson et al., USA, 2015 [111]	Case control	11 -- gestational HTN (10/11 with PE)	At delivery
		37 – healthy pregnant controls	
Baker et al., 2010 [80]	Nested Case Control	51 – developed PE	2nd trimester
		204 – healthy pregnant controls	
Bodnar et al., USA, 2007 [18]	Nested Case Control	55 – developed PE	<22 weeks
		219 – healthy pregnant controls	
Bodnar et al., USA, 2014 [79]	Case Control	717 – developed PE	<26 weeks gestation
		*560 mild; 157 severe*	
		2986 – healthy ﻿pregnant﻿controls	
Gidlof S, et al. Sweden, 2015 [112]	Nested Case Control	39 – developed preeclampsia	12th week of gestation
		120 – healthy pregnant controls	
Halhali, Mexico, 2004 [95]	Case Control	10 -- developed PE, 40 – healthy pregnant controls	Median 20.7 weeks gestation
Halhali et al., Mexico, 2007 [113]	Case Control	26 -- women with PE 26 – healthy pregnant controls	3rd trimester
Lechtermann C, et al. Germany, 2–14 [85]	Case Control	20 – women with PE	At delivery
		43 – healthy pregnant controls	
Mohaghegh et al., Iran, 2015 [89]	Case Control	41 -- women with PE 50 – healthy pregnant controls	Time of labor
Powe, USA, 2010 [86]	Nested Case Control	39 -- developed PE 131 – healthy pregnant controls	1st trimester
Robinson et al., USA, 2010 [25]	Case Control	50 -- women with EOSPE	29 weeks gestation
		100 – healthy pregnant controls	
Schneuer et al., Australia, 2014 [114]	Nested Case Control	5109 pregnant women (223 with PE and 29/223 with EOSPE)	10–14 weeks gestation
Singla et al., India, 2015 [87]	Case Control	74 -- nulliparous women with PE	>30 weeks gestation
		100 -- healthy nulliparous controls	
Ullah et al., Bangladesh, 2013 [82]	Case Control	33 -- women with PE	>20 weeks gestation
		79 – normal pregnancy controls	
Wetta et al., UK, 2013 [96]	Case Control	100 -women with PE	15–21 weeks gestation
		200 – healthy pregnant controls	
Woodham et al., USA, 2011 [15]	Nested Case Control	41 – women with severe PE	2nd Trimester
		121 – uncomplicated birth controls	
Xu et al., USA, 2014 [53]	Case Control	100 – women with PE	≤ 24 weeks gestation
		100 – uncomplicated birth controls	
Yu et al., UK, 2012 [115]	Case Control	60 –late PE	11–13 weeks gestation
		30 –early PE	
		1000 – healthy controls	
Retrospective Cohort Study			
Alvarez-Fernandez et al., Spain, 2014 [90]	Retrospective Cohort	257 -- women attending obstetric triage with suspicion of PE	1st Trimester and 20 weeks of gestation
Scholl et al., USA, 2013 [78]	Retrospective cohort	1141 -- low income and minority pregnant women	Entry to care (mean 13.7 ± 5.7 weeks)
Prospective Cohort Studies			
Burris et al., USA, 2014 [94]	Prospective Cohort	1591 -- pregnant women	16.4–36.9 weeks gestation
Haugen et al., Norway, 2000 [77]	Prospective Cohort	23,423 -- pregnant women	Vitamin D intake at weeks 15, 22, and 30 gestation
Shand et al., Canada, 2010 [37]	Prospective cohort	221 -- women at risk for PE	10–20 weeks gestation
Wei et al., Canda 2012 [60]	Prospective Cohort	697 -- pregnant women receiving vitamin C and E supplementation for the prevention of PE	12–18 weeks gestation, 24-26 weeks gestation
Wei, Canada, 2013 [14]	Prospective Cohort	697 -- pregnant women receiving vitamin C and E supplementation for the prevention of PE	24–26 weeks gestation
Zhou, China, 2014 [116]	Prospective Cohort	74 – women with PE	16–20 weeks gestation

**Table 3:** Association between vitamin D and pre-eclampsia (PE) in observational studies

Author, Location, Year	Key Findings	Results
August et al., USA, 1992 [88]	↓ 1,25OH2D in women with PE vs chronic HTN and normal women	1,25 OH2D levels: PE: 37.8 +/− 15 pg/ml chronic HTN: 75 +/− 15 pg/ml (*p* < 0.05); normal women: 65 +/− 10 pg/ml (*p* < 0.05)
Fernandez- Alonzo et al., Spain, 2012 [97]	↔ PE and 25(OH)D levels	25(OH)D: <49.9 nmol/L: 28.6% (2/7 women); 49.9–74.9 nmol/L: 42.9% (3/7 women); ≥ 74.9 nmol/L: 28.6% (2/7 women) (*p* = 0.91)
Pena HR, et al. Brazil 2015 [83]	↑ frequency of 25(OH)D deficiency <20 ng/mL in PE compared to healthy non obese controls	PE: 52.1% (25 women) Non obese controls: 14.9% (7 women) (*P* = 0.0006)
Abedi et al., Iran, 2014 [84]	↑ vitamin D deficiency (<25.0 nmol/L) in PE cases	OR = 24.04 95% CI: 2.14–285.4
Achkar et al., Canada, 2015 [81]	↑ PE in women with 25(OH)D < 30 nmol/L vs women with at least 50 nmol/L	Adjusted OR: 2.23 95% CI: 1.29–3.83
Anderson et al., USA, 2015 [111]	↔ proportion of women with inadequate <30 ng/mL 25(OH)D levels in HTN group vs control group	73% (HTN/PE group) vs 69% (control group) (*p* = 0.22)
Baker et al., 2010 [80]	↑ Severe PE in women with 25(OH)D < 50 nmol/L compared to levels of at least 75 nmol/L	Adjusted OR: 5.41 95% CI: 2.02–14.52 (*P* = 0.001)
Bodnar et al., USA, 2007 [18]	↑ PE in women with 25(OH)D < 37.5 nmol/L compared to levels of >37.5 nmol/L	Adjusted OR: 5.0 95% CI: 1.7–14.1
Bodnar et al., USA, 2014 [79]	↓ Severe PE in women with 25(OH)D ≥ 50 nmol/L compared to levels <50 nmol/L	Adjusted RR: 0.65 95% CI: 0.43–0.98
Gidlof S, et al. Sweden, 2015 [112]	↔ 25(OH)D levels in PE and healthy controls; ↔ 25(OH)D deficiency < 50 nmol/L in PE and controls	25(OH)D: PE: 52.2 ± 20.5 nmol/L; Controls: 48.6 ± 20.5 nmol/ L (*p* = 0.3); 25(OH)D deficiency: PE: 38%, Controls: 51.7% (*p* = 0.1)
Halhali, Mexico, 2004 [95]	↔ 1,25(OH)_2_D in women before they developed PE	1,25(OH)_2_D: median (interquartile range) PE: 31 pg/mL (26–34) NT: 29 pg/mL (24–36) (*p* = 0.44)
Halhali et al., Mexico, 2007 [113]	↓ 1,25(OH)_2_D levels in women with PE vs controls	25(OH)D: PE: 486.7 ± 167.2 nmol/L Controls: 731.1 ± 262.1 nmol/L (*p* < 0.05)
Lechtermann C, et al. Germany, 2–14 [85]	↓ 25(OH)D levels in PE in summer compared to controls, 1,25(OH)_2_D ↓ only in winter	25(OH)D: PE:18.2 ± 17.1; Control: 49.2 ± 29.2 ng/mL, (*P* < 0.001); 1,25(OH)_2_D: 291 ± 217 vs 612.3 ± 455 pmol/mL (*P* < 0.05)
Mohaghegh et al., Iran, 2015 [89]	↓ mean 25(OH)D in PE compared to pregnant controls without PE	25(OH)D: PE: 37.9 ± 33.9 nmol/L Controls: 58.2 ± 38.2 nmol/L (*p* = 0.001)
Powe, USA, 2010 [86]	↔ women with PE and controls with 25(OH)D < 15.0 nmol/L	Adjusted OR: 1.35 95% CI: 0.40 to 4.50
Robinson et al., USA, 2010 [25]	↑ EOSPE in women with maternal 25(OH)D levels <=19.6 nmol/L compared to levels >19.6 nmol/L	OR: 3.60 95% CI: 1.71–7.58 (*p* < 0.001)
Schneuer et al., Australia, 2014 [114]	↔ PE or EOSPE and low 25(OH)D (< 25 nmol/L)	Adjusted OR- all PE: 0.46 95% CI: 0.19–1.10 Adjusted OR- EOSPE: 1.40 95% CI: 0.20 to 9.89
Singla et al., India, 2015 [87]	↓ mean serum vitamin D in women with PE vs controls	PE: 24.2 ± 12.4 nmol/L Controls: 36.9 ± 16.7 nmol/L; (*p* = 0.0001)
Ullah et al., Bangladesh, 2013 [82]	↑ PE per 25 nmol/L decrease in 25(OH)D level	Adjusted OR: 1.66 95% CI: 1.05–3.02
Wetta et al., UK, 2013 [96]	↔ between PE and 25-OH D < 30 ng/mL and <37.4 nmol/L	<30 ng/mL Adjusted OR: 1.1 95% CI: 0.6–2.0 Adjusted OR: 1.4 (<37.4 nmol/L) 95% CI: 0.7–3.0
Woodham et al., USA, 2011 [15]	↓ Severe PE in women with 10 nmol/L increase in maternal 25(OH)D level	Adjusted OR: 0.62 95% CI: 0.51–0.76
Xu et al., USA, 2014 [53]	↑ PE in women with vitamin D deficiency (<37.5 nmol/L)	OR: 4.4 95% CI: 1.8–10.8
Yu et al., UK, 2012 [115]	↔ serum vitamin D raw values in PE and controls	25(OH)D levels: Controls: 46.8 nmol/L (27.8–70.0;) Early PE: 32.2 nmol/L (22.7–50.4); Late PE: 39.2 nmol/L (22.1–63.0) (*P* = 0.231)
Alvarez-Fernandez et al., Spain, 2014 [90]	↑ PE in women with 25(OH)D levels <50 nmol/L compared to levels >50 nmol/L after 20 weeks of gestation	OR: 4.6 95% CI:1.4–15 (*P* = 0.010)
Scholl et al., USA, 2013 [78]	↑ PE in women with 25(OH)D < 49.9 nmol/L and hyperparathyroidism	Adjusted OR: 2.86 95% CI: 1.28–6.41
Burris et al., USA, 2014 [94]	↔ PE and 25(OH)D levels compared at each 25 nmol/L increase in 25 (OH)D	Adjusted OR: 1.14 95% CI: 0.77–1.67
Haugen et al., Norway, 2000 [77]	↓ PE in women taking 10–15 mcg/d as compared with no supplements	Adjusted OR: 0.73 95% CI: 0.58–0.92
Shand et al., Canada, 2010 [37]	↔ PE and 25(OH)D levels <37.5 nmol/L	Adjusted OR: 0.91 95% CI: 0.31–2.62
Wei et al., Canda 2012 [60]	↔ PE and 25(OH)D < 50 nmol/L	Adjusted OR: 1.24 95% CI: 0.58–2.67 (*p* = 0.58)
Wei, Canada, 2013 [14]	↑ PE and women with ↓ PIGF levels and maternal 25(OH)D < 50 nmol/L	Adjusted OR: 2.97 95% CI: 1.23–7.20
Zhou, China, 2014 [116]	↔ PE and 25(OH)D levels	25(OH)D levels Group A (*n* = 13): 41.4 ± 6.5 nmol/L; Group B (*n* = 36): 62.1 ± 7.0 nmol/L; group C (*n* = 25): 89.6 ± 13.0 nmol/L; (*p* = 0.900)

A case-cohort study of women from 12 different United States (US) sites whose vitamin D levels were measured at ≤26 weeks of gestation showed that 25(OH)D levels greater than 50 nmol/L were associated with a 40% reduction in risk for severe PE (0.65 [95% CI 0.43 to 0.98]), although there was no reduction in absolute and relative risk for the milder clinical subtypes of PE when 25(OH)D levels were greater than 50 nmol/L [79]. In a nested case control study of 274 nulliparous pregnant women conducted previously by the same investigator, there was an OR of 5.0 for PE in early pregnancy (<22 weeks) when maternal 25(OH)D was less than 37.5 nmol/l after controlling for education in addition to the common confounders (95% CI: 1.7–14.1). Interestingly, it was reported that newborns of pre-eclamptic mothers were more than twice as likely to have 25(OH)D levels less than 37.5 nmol/L (aOR = 2.2, 95% CI: 1.2–4.1) than newborns of healthy controls [18]. Another nested case control study of 225 women with singleton pregnancies reported an OR of 5.41 for severe PE among women with mid-gestation vitamin D deficiency after controlling for multi-parity (95% CI: 2.02–14.52) compared to women with vitamin D levels of at least 75 nmol/L [80]. A larger Canadian case control study reported a more than twice as likely odds for PE in women with 25(OH)D less than 30 nmol/L compared to women with at least 50 nmol/L (95% CI: 1.29–3.83). There was a dose response relationship between maternal 25(OH)D and risk of PE with a threshold of effect at 50 nmol/L [81].

Observational studies which measured vitamin D status after the onset of PE [53, 82] near delivery [83] or at delivery [84, 85] suggest an inverse association with PE. A US case control study reported a trend toward increased risk of PE with 25(OH)D levels less than 15.0 nmol/L (OR = 2.5 [95% CI: 0.89-6.9]) when compared to the controls (chosen randomly from among women who remained normotensive throughout pregnancy, and did not have gestational diabetes mellitus or gave birth to SGA infants). However, this trend was not significant after adjusting for BMI and other covariates. The investigators observed a trend towards increased risk of PE at very low levels of 25(OH)D, suggesting that there may be an association at the low extreme [86]. A recent North Indian case control study of nulliparous women with PE and singleton pregnancies reported serum vitamin D to be significantly lower among PE cases vs. controls at the time of delivery (24.2 +/− 12.4 nmol/L, 36.9 +/− 16.7 nmol/L, respectively; *p* = 0.0001). Similar vitamin D levels were found in women with mild and severe PE [87]. Two cross-sectional studies report 25(OH)D and 1,25(OH)_2_D levels to be lower in women with PE in the third trimester. Although these studies find an inverse association between vitamin D levels and PE, this association may be confounded by the gestational age at serum collection. These studies are also limited in that odds ratios are not reported [83, 88].

Correlation between low serum vitamin D in women with established PE was also found in studies in which populations had specific risk factors for vitamin D deficiency such as race or seasonal sun exposure. A nested case control study showed an association between EOSPE and vitamin D deficiency after the diagnosis of EOSPE. Serum 25(OH)D was measured at the time of diagnosis of EOSPE (~ 29 weeks of gestation). Controls were matched to cases according to gestational age at diagnosis with EOSPE and race. In patients with EOSPE (*n* = 50), 25(OH)D was significantly lower compared to the controls (*n* = 100) (44.9 vs. 79.9 nmol/L; *p* < 0.001). There was an adjusted odds ratio (aOR) of 3.6 [(95% CI 1.71–7.58), *p* < 0.001] for EOSPE when maternal 25(OH)D was less than or equal to 19.6 nmol/L. There was also a 12-fold increase in odds of diagnosis with EOSPE in African American women, who had the lowest mean 25(OH)D concentration among groups categorized by race [25]. In an Iranian case control study conducted in the fall and winter months, 25(OH)D levels were measured at the time of delivery in 41 pre-eclamptic women, 50 normal women and from their umbilical cord samples immediately after birth. This study found mean 25(OH)D levels to be significantly lower in pre-eclamptic women versus normal women (37.9 ± 33.9 nmol/L vs. 58.2 ± 38.2 nmol/L, respectively, *p* = 0.001). There was a significant relationship between vitamin D levels in pre-eclamptic women with levels in their neonates (*r* = 0.901, *p* = 0.0001) [89].

Two studies of vitamin D in PE cases have evaluated the possible relationship with angiogenic factors. In a small nested case control study that matched cases of severe PE by race and ethnicity to healthy controls, 25(OH)D levels were found to be an independent predictor for severe PE. In women with a 10 nmol/L increase in maternal 25(OH)D, there was a 38% reduction in the odds of severe PE (aOR = 0.62, 95% CI: 0.51–0.76). Women with severe PE had significantly lower levels of PIGF (*p* = 0.03) and VEGF (*p* = 0.0007) and a higher sFLT-1/PlGF ratio (*p* = 0.02) compared to controls. However, there were no independent correlations reported between 25(OH)D levels and these angiogenic markers. A limitation of this study was heterogeneity of parity, a potentially confounding variable, in pre-eclamptic cases (44% multiparous) [15]. A retrospective fully blinded cohort study of 257 pregnant women in an obstetric emergency service in Spain also found no association between SFlt-1/PIGF and 25(OH)D levels in women with PE. However, this study found a greater risk of late-onset PE among women with low 25(OH)D level (<50 nmol/L) (OR 4.6, 95% CI 1.4–15) and increased risk of both early- and late-onset PE when sFlt-1/PIGF ratios were above corresponding cut-points (ORs 58 [95% CI 11–312] and 12 [95% CI 5.0–27], respectively) [90].

There have been a small number of clinical trials studying associations between maternal vitamin D and hypertensive disorders in pregnancy (Table 4). In a 50-year-old controlled trial of 5644 women, Olsen & Secher assessed the preventive effect of a multi-vitamin mineral supplement, which included iron, calcium, iodine, manganese, copper, vitamin B complex, vitamin C, and 260 mg/day of calcium and halibut liver oil containing 2500 IU/g vitamin D per day. Among women who were given supplementation from week 20 of gestation onwards, there was a 31.5% reduction in the odds of PE (*p* < 0.005). No significant effect was observed in the odds of developing gestational hypertension [91]. Of note, omega-3 fatty acids found along with vitamin D in cod liver oil are likely to play an independent role in preventing PE [92, 93].

**Table 4:** Association of vitamin D supplementation and pregnancy-induced hypertension (PIH) outcomes in clinical trials

Author, Location, Year	Study Design	Subjects	Intervention	Key Findings	Results
Olsen and Secher, Denmark, 1990 [91]	Randomized controlled clinical trial	5644 pregnant women	2500 IU vitamin D supplement versus no supplement at week 20 of pregnancy	↓ PE	31.5% reduction in the odds (*p* < 0.005)
Olsen et al., Europe, 2000 [98]	Randomized placebo controlled clinical trial	386 women who previously experienced PIH	2.7 g n-3 fatty acids/day given from 33 weeks until delivery vs olive oil placebo	↔ PIH recurrence risk	OR = 0.98 95% CI: 0.63–1.53

### Evidence that suggests no association between maternal vitamin D deficiency and PE

While the preceding studies support a link between vitamin D deficiency during pregnancy and risk of PE, some studies present conflicting evidence (Tables 2 and 3). Most of these studies were observational, and measured maternal vitamin D concentration before the diagnosis of PE as opposed to measuring at the time of diagnosis or delivery.

A 2010 prospective cohort study did not find an association between low serum 25(OH)D levels (<37.5 nmol/L) in the first half of pregnancy and the development of PE (aOR = 0.91 CI: 0.31–2.62) or gestational hypertension (aOR = 1.55 CI: 0.58–4.17) in women who were at high-risk for PE after controlling for smoking and parity. This study used high-risk women, both nulliparous and multiparous, who did not develop PE as controls [37]. Another cohort study in 2014 found no association between low plasma 25(OH)D concentration (<25 nmol/L) and PE in women at 16.4–36.9 weeks of gestation (aOR = 0.60 95% CI:0.14–2.56). Rather, investigators found for every 25 nmol/L increase in 25(OH)D, the aOR for PE was 1.14 (95% CI, 0.77–1.67) [94].

In 2012, Wei et al. retrospectively analyzed vitamin D status and the risk of PE in 697 nulliparous women with singleton pregnancies in a randomized, placebo-controlled trial of Vitamin C and E supplementation for the prevention of PE. After controlling for smoking, the study showed that in the first trimester (mean 11 weeks), vitamin D deficiency was not significantly associated with an increased risk of PE (aOR = 1.24 95% CI: 0.58–2.67; *p* = 0.58) [60]. In a 2013 follow-up study of women from the same cohort, Wei et al. found that PIGF level was inversely associated with PE (*p* < 0.05) [13]. In a multivariate logistic model to control for PIGF there was an aOR of 2.97 (95% CI: 1.23–7.20) for PE among women with 25(OH)D levels less than 50 nmol/l. This was only an 8.3% reduction in the risk for PE compared to when PIGF levels were not considered. There was no statistical evidence of interaction between PIGF and vitamin D (*p* = 0.54) [14].

In 2004, a longitudinal case control study did not find altered circulating 1,25(OH)_2_D during mid-pregnancy in those who developed PE compared to healthy, normotensive women [95]. In 2010, Powe et al. suggested that earlier studies have measured total 25(OH)D as a vitamin D/Vitamin D Binding Protein (VDP) complex, and not the free vitamin. Therefore, Powe et al. conducted a study measuring levels of both total and free 25(OH)D in the first trimester of PE and normotensive pregnancies. They found total and free 25(OH)D levels were similar in cases and controls (68.4 ± 4.7 nmol/L versus 71.9 ± 2.0 nmol/L, *p* = 0.435). There was no association between 25(OH)D levels <15.0 nmol/L and risk of PE (aOR = 1.35 95% CI: 0.40 to 4.50) [86]. In a nested case control study of women with singleton pregnancies, 100 pregnant women with PE were matched to 200 healthy controls who delivered at 39–40 weeks. 25(OH)D levels were assessed from stored blood samples drawn between 15 and 21 weeks gestation for multiple marker screening. After adjusting for covariates, vitamin D insufficiency and deficiency were not significantly associated with PE (aOR = 1.1 95% CI: 0.6–2.0; aOR = 1.4 95% CI: 0.7–3.0, respectively) [96].

In a cross-sectional study of 466 pregnant women attending an outpatient clinic in Spain, serum 25(OH)D was measured between 11 and 14 weeks of pregnancy. No associations were found between maternal 25(OH)D less than 49.9 nmol/L or 25(OH)D between 49.9 and 74.9 nmol/L in cases of PE and gestational hypertension identified during follow-up [97].

In 2000, a double-blind, randomized, placebo-controlled trial evaluated the effects of 2.7 g fish oil supplementation given prophylactically from 20 weeks until delivery in women who had previously experienced Pregnancy Induced Hypertension (PIH) (*n* = 386). Supplementation with fish oil did not affect the recurrence risk for PIH in the prophylactic trial (OR = 0.98 CI: 0.63–1.53). Vitamin D concentrations were not reported [98].

## Discussion

Observational studies evaluating the association between vitamin D and PE have shown inconsistent results and must be interpreted cautiously. This may be a result of issues with study design and methodology, including lack of adjustment of key confounding variables and methods of measuring vitamin D levels.

Study findings must be interpreted in the context of study design. Most previous studies were based on cohort studies that collected clinical data and stored blood in repositories at regular intervals. Many researchers utilized a case-control study design within these cohorts, or performed cross-sectional studies. Residual confounding and differences between groups may explain the association between vitamin D deficiency and PE.

Most studies controlled for maternal age, body mass index, season, and gestational trimester at sample collection. In addition, some studies also controlled for smoking [14, 37]. Smoking has consistently been shown to reduce the risk of PE and gestational hypertension [99, 100]. This could be due to an association between smoking and lower circulating concentrations of anti-angiogenic proteins and higher concentration of pro-angiogenic proteins [101]. Smoking has also been linked to lower vitamin D concentrations [37]. Smoking may be an important confounder and should be considered in studies linking vitamin D to PE. The pathophysiology of PE may also vary by parity [102]. Wetta et al., [96], Shand et al., [37] and Bodnar et al., [18] have controlled for parity in their studies.

Individuals receive the majority of their vitamin D from sunlight, linking seasonality to the development of PE. Seasonality is also considered a confounder, particularly in studies related to causal effects of vitamin D on PE. Seasonal and latitudinal variation has an effect on vitamin D_3_ production in the skin [103]. According to a study by Magnus and Eskild [104], in Norway, mothers of children born in August had the lowest risk of PE. Risk in this study was highest in the winter months (for December, aOR: 1.26, 95% CI: 1.20–1.31) [104]. Similarly, Bodnar et al. [18] found that the incidence PE among white women in the United States was highest in the winter, when production of cutaneous vitamin D_3_ is limited in temperate zones and serum 25(OH)D are at their lowest levels. However, despite this known association, not all studies looking at PE as an outcome report information on seasonal, latitudinal variation, sun exposure and lifestyle differences—all of which may differentially expose individuals to sunlight in the sample population.

Maternal dietary intake of vitamin D from foods or supplements may also vary. Oily fish and cod liver oil (n-3 fatty acids) are a rich source of vitamin D. In the Norwegian diet, intake of vitamin D is correlated with intake of long chain n-3 fatty acids [77]. The use of cod liver oil as a food supplement in some diets presents a challenge in determining an isolated effect of vitamin D supplementation. This was noted by Haugen et al. [77], who were unable to control for the intake of fatty acids in their analysis. In their secondary analysis with intake of long chain n-3 fatty acids and vitamin D, a weaker association with the incidence of PE was observed [77].

Although randomized clinical trials (RCTs) offer the opportunity to design studies with power to provide definitive evidence, few have been performed in this field. RCTs reviewed in this article were not able to study vitamin D supplementation independently of calcium and/or other multivitamin/micronutrient supplementation. In a Cochrane review of Vitamin D supplementation for women during pregnancy, women who received vitamin D and calcium supplementation had a lower risk of pre-eclampsia than those not receiving any intervention (RR 0.51; 95% CI 0.32 to 0.80; three trials, 1114 women, moderate quality), yet an increased risk for preterm birth (RR = 1.57; 95% CI: 1.02–2.43; three studies, 798 women, moderate quality). In trials with an intervention of vitamin D without calcium, women who received vitamin D supplements had a statistically nonsignificant lower risk of pre-eclampsia than those receiving no intervention or placebo (8.9% versus 15.5%; risk ratio (RR) 0.52; 95% CI 0.25 to 1.05; low quality) with no adverse outcomes [105]. The role of fish oils in PE remains uncertain and similarly poses a challenge in determining an isolated effect of vitamin D supplementation, unless studies are designed such that they consider this. In the RCTs reviewed, no conclusions can be made regarding the independent effects of vitamin D in preventing or treating PE.

Studies must also be interpreted according to how vitamin D exposure is defined and measured. “The free hormone hypothesis” postulates that hormones that are free from their binding proteins may enter cells to perform biological functions [106]. During pregnancy, VDBP increases by 2-fold [86]. Future investigations should consider concurrently measuring VDBP levels and calculated free vitamin D levels when considering the role of vitamin D in PE [18]. Additionally, investigators should attempt to assess preconception and early pregnancy dietary intake, sun exposure or baseline vitamin D status.

Studies that find a role early in pregnancy, before the clinical onset of PE, suggest that vitamin D may play a role in the modulation of a pro-inflammatory response or regulation of angiogenic factors. It is possible that biomarkers for PE, such as angiogenic factors VEGF and PIGF, may serve as mediators in the pathway linking vitamin D metabolites to PE. Despite evidence that low vitamin D levels increase risk for PE early in pregnancy, very few studies have tried to relate this effect with the regulation of angiogenic or anti-angiogenic factors. Although current evidence supports an association between angiogenic factors and PE, these studies have not found an association between vitamin D levels and angiogenic factors [14, 15]. Studies with longitudinal study design carefully controlling for temporal sequences of changes in vitamin D levels in women with PE are needed to identify the role of vitamin D and any potential mediators in the pathway linking vitamin D to PE.

There are important clinical considerations that necessitate the need for further research on this topic. There is limited data on the most efficacious dose of vitamin D to prevent pre-eclampsia while avoiding toxicity. The doses of vitamin D supplements in the studies reviewed ranged from 400 to 2500 IU daily [91]. These studies reported no major adverse effects of supplementation at these doses [107–109]. Recently, participants in the Vitamin D for Antenatal Asthma (VDAART) randomized double-blind placebo controlled trial took a daily dose of 4000 IU vitamin D supplementation plus a multivitamin with 400 IU vitamin D or placebo (placebo pill plus a multi-vitamin with 400 IU vitamin D daily) to assess the effect of vitamin D supplementation on the frequency of PE among pregnant women with a high risk for atopic disease [110].

The investigation by Haugen et al. suggests a potential for a role for the frequency and timing of vitamin D supplementation and risk for PE, given that the total intake greater than 800 IU/day did not reduce risk. However, Haugen et al. found that women who took supplements at all three points of pregnancy (before pregnancy, early pregnancy and in late pregnancy) were at a lower risk for PE compared with women who took supplements in only early or late pregnancy or did not take any supplements [77]. In the VDAART, frequency of PE among pregnant women with a high risk for atopic disease was not lower when a supplementation of 4400 IU vitamin D daily was given in early pregnancy (10–18 weeks). However, women with a serum vitamin D status of ≥75 nmol/L that was maintained from enrollment through late pregnancy had a significantly lower risk of PE versus women who had <75 nmol/L (*P* = 0.04). But this vitamin D level was maintained in only 74% of pregnancies in the supplementation group by weeks 32 to 38 of gestation, suggesting that supplementation at even earlier time-points or before pregnancy may be necessary to maintain sufficient vitamin D levels that are protective against PE. This was further supported by peripheral blood gene expression patterns relating to immune and inflammatory processes identified as early as the 10th week of pregnancy that were unique in women who went on to develop PE [110].

Additionally, early-onset and late-onset PE have unique clinical features, biomarkers and prognoses that may guide clinical dose recommendations. Studies reported varying degrees of risk for clinical subtypes of PE among women with low vitamin D levels [79, 90]. In order to guide the clinical recommendations, investigators must use a standard set of definitions for the disease and clinical subtypes in future research. In the current literature, the definition of PE was not consistent across studies, limiting our ability to draw generalizations. For example, clinical cutoffs for proteinuria varied slightly among studies [86, 87]. Replication of findings using a uniform set of definitions for PE and clinical subtypes can inform the use of vitamin D supplementation for the prevention of PE.

Robinson et al. [25] suggest that vitamin D deficiency may be a factor in the disproportionate incidence of adverse pregnancy outcomes in African American women since this group had the lowest vitamin D levels among the populations they studied. While some studies have been able to capture an adequate representation of African American women [96], there is a need for further research on populations with very low vitamin D levels to understand this observation and identify factors that predispose high-risk groups to PE and its more threatening clinical subtypes.

Despite the clear association of serum deficiency with PE, a better understanding of the variable impact of supplementation is needed to identify potential of genetic and environmental interactions, as well as pre-conception data to identify the critical time window for therapeutic potential of vitamin D, if any.

## Conclusion

There is consistent evidence of an association between low vitamin D concentrations and adverse PIH outcomes. Results from vitamin D supplementation during pregnancy did not show a statistically significant independent effect of vitamin D on the risk of PE and PIH.

At this time, our understanding of an ideal supplementation dose of vitamin D to reduce PIH remains incomplete. Future studies should include follow up from the pre-conception period until delivery to elucidate the mechanisms and interactions which drive vitamin D status, response, and onset of PE to inform population-specific dose recommendations.

## Additional file

Additional file 1: PRISMA Checklist. Preferred Reporting Items for Systematic Reviews and Meta-Analyses: PRISMA. (DOC 63 kb)

## Abbreviations

1,25(OH)_2_D: 1,25-dihydroxyvitamin D
25(OH)D: 25-hydroxyvitamin D
EOSPE: Early Onset Severe Pre-eclampsia
HELLP: Hemolytic anemia, Elevated liver enzymes, Low Platelets
IL-10: Interleukin-10
IUGR: Intrauterine Growth Restriction
LOSPE: Late Onset Severe Pre-Eclampsia
PE: Pre-eclampsia
PIGF: Placental Growth Factor
PIH: Pregnancy Induced Hypertension
PTH: Parathyroid Hormone
RAS: Renin Angiotensin System
RTC: Randomized Controlled Trial
sFLT-1: Soluble Fms-like Tyrosine Kinase 1
SGA: Small for Gestational Age
VDBP: Vitamin D Binding Protein
VDR: Vitamin D Receptor
VEGF: Vascular Endothelial Growth Factor
VSMC: Vascular Smooth Muscle Proliferation
